# Evolutionary Plasticity and Innovations in Complex Metabolic Reaction Networks

**DOI:** 10.1371/journal.pcbi.1000613

**Published:** 2009-12-18

**Authors:** João F. Matias Rodrigues, Andreas Wagner

**Affiliations:** 1 Department of Biochemistry, University of Zürich, Zürich, Switzerland; 2 The Santa Fe Institute, Santa Fe, New Mexico, United States of America; 3 The Swiss Institute of Bioinformatics, Zürich, Switzerland; University of Illinois at Urbana-Champaign, United States of America

## Abstract

Genome-scale metabolic networks are highly robust to the elimination of enzyme-coding genes. Their structure can evolve rapidly through mutations that eliminate such genes and through horizontal gene transfer that adds new enzyme-coding genes. Using flux balance analysis we study a vast space of metabolic network genotypes and their relationship to metabolic phenotypes, the ability to sustain life in an environment defined by an available spectrum of carbon sources. Two such networks typically differ in most of their reactions and have few essential reactions in common. Our observations suggest that the robustness of the *Escherichia coli* metabolic network to mutations is typical of networks with the same phenotype. We also demonstrate that networks with the same phenotype form large sets that can be traversed through single mutations, and that single mutations of different genotypes with the same phenotype can yield very different novel phenotypes. This means that the evolutionary plasticity and robustness of metabolic networks facilitates the evolution of new metabolic abilities. Our approach has broad implications for the evolution of metabolic networks, for our understanding of mutational robustness, for the design of antimetabolic drugs, and for metabolic engineering.

## Author Summary

Understanding the fundamental processes that shape the evolution of bacterial organisms is of general interest to biology and may have important applications in medicine. We address the questions of how bacterial organisms acquire innovations, including drug resistance, allowing them to survive in new environments. We simulate the evolution of the metabolic network, the network of reactions that can occur inside a living organism. The metabolic network of an organism depends on the genes contained in its genome and can change by gaining genes from other organisms through horizontal gene transfer or loss of gene activity through mutations. Our observations suggest that the robustness to gene loss in *Escherichia coli* is typical of random viable metabolic networks of the same size. We also find that metabolic networks can change significantly without causing the loss of an organism's ability to survive in a given environment. This property allows organisms to explore a wide range of novel metabolic abilities and is the source of their ability to innovate. Finally we present a method to find reactions that are essential across all organisms. Drugs targeting such a reaction may avoid drug resistance mutations that bypass the reaction.

## Introduction

Organisms, especially microbes, thrive on organic nutrients with bewildering diversity: the vast majority of organic molecule can mean “food” for some species. From a microbe's perspective, acquiring the ability to survive on a new carbon source can make the difference between life and death; such an acquisition can thus be an important evolutionary innovation. We here study the properties of metabolic systems that facilitate such innovations.

**Figure 1:**
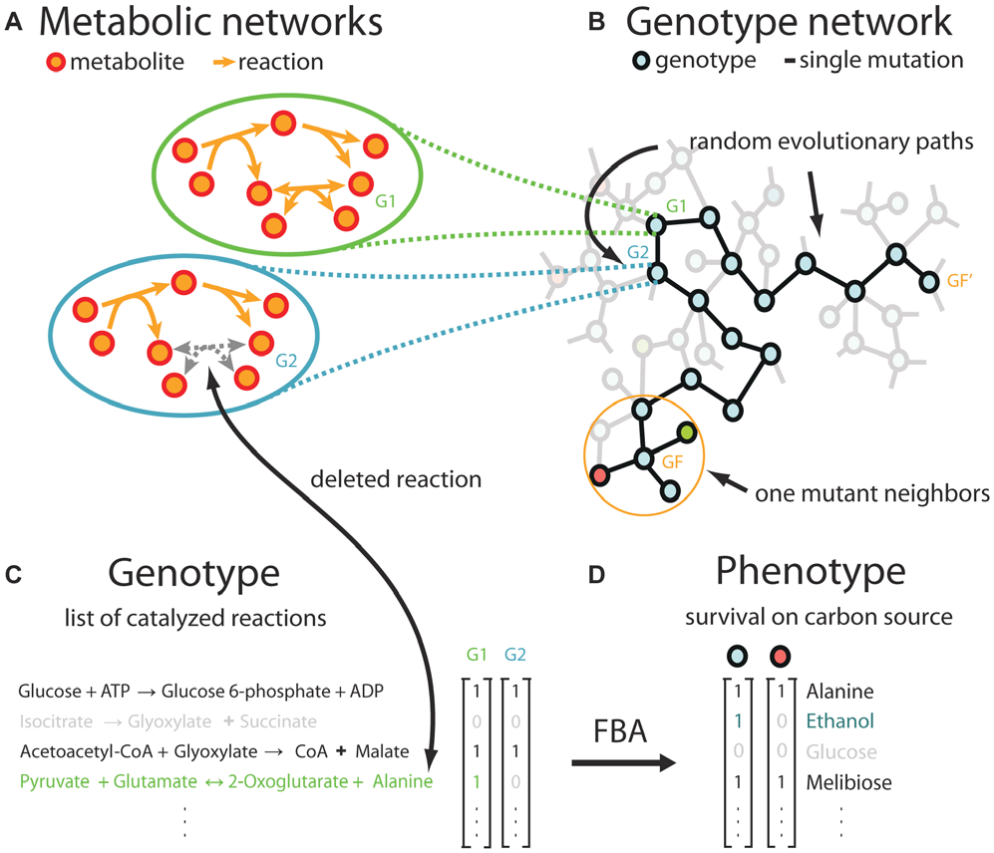
Exploration of a vast genotype space of metabolic networks. A genotype can be represented in different ways: (A) as a metabolic network, (B) as a node in a genotype network, or (C) as a binary vector listing the reactions catalyzed. Genotypes on the genotype network (B) that are connected differ by only one mutation. The color of the genotype circles indicates their metabolic phenotype. Metabolic phenotypes are computed using FBA applied to 101 environments with different carbon sources. They can be represented as a binary vector listing the environments a genotype is viable in (D). Random evolutionary walks can be seen as paths on a genotype network. Two independent random walks are shown with the same starting genotype (G1) and two final genotypes (GF and GF'), passing through intermediate genotypes (i.e.: G2) that differ by one mutation. Mutations are chosen at random. They can be additions or deletions of individual reactions from the corresponding metabolic network but they must not change the phenotype. The neighborhood of each genotype can be analyzed by characterizing the phenotype of the one mutant neighbor genotypes (approximately 5’800 neighbors per genotype). The number of genotypes in the genotype space is 2^5800^. Each genotype is able to catalyze approximately 1000 out of 5800 possible reactions.

The evolution of biological macromolecules has received serious attention for decades [1]. The same is not true for biological systems on higher levels of organization, such as regulatory and large complex metabolic networks. One reason is a comparative paucity of data for such networks. Another reason is the inherent difficulty in characterizing both network genotypes and network phenotypes. Recent work on genome-scale metabolic networks reduces these limitations. First, metabolic genotypes have recently been characterized for several model organisms [2]–[4]. Second, databases of metabolic reactions inform us about a broad spectrum of chemical reactions catalyzed by enzymes in living things. Third, flux balance analysis [5] allows us to compute metabolic phenotypes from metabolic genotypes (Figure 1). Taken together, these developments allow us to study the evolution of metabolic networks in greater depth.

The functions and phenotypes of biological macromolecules are robust to genetic change. Such robustness has important implications for the evolutionary plasticity of molecules, the ability of molecules to evolve new properties. Through mutations that do not affect a molecule's function, vast regions of phenotype space can be explored, regions in which molecules with novel phenotypes can lie [1],[6]. Does the same hold for genome-scale biological networks? Can biological networks with similar phenotypes have a vast number of interconnected and different genotypes, thus being both highly robust and having large evolutionary plasticity? These questions currently have few answers. We study the evolution of genome-scale metabolic networks to provide such answers.

For our purpose, a metabolic *genotype* is a set of chemical reactions – catalyzed by gene-encoded enzymes – that take place in an organism. Any one organism's metabolic network exists in a much larger space of metabolic genotypes. This space is defined by the biochemical reactions known to be realized in living cells. Any one organism's genotype can be thought of as a point in this space, where some biochemical reactions occur and others are absent. Genotypes can thus be represented as binary strings whose entries indicate presence (‘1’) or absence (‘0’) of reactions (Figure 1) in an organism. We define the *phenotype* of such a network as its ability to sustain life in a given environment or set of environments. This means that the network must be able to produce all biochemical precursors (amino acids, nucleotides etc.) that are necessary to allow a free-living heterotrophic organism such as *Escherichia coli* to grow from environmental resources. We here consider 101 minimal environments that only differ in their carbon source. Specifically, these environments provide only a terminal electron acceptor (O_2_), a source of nitrogen (NH_3_), sulfate (SO_4_), phosphate (PO_4_), and one out of 101 sources *C* of carbon (see Text S1 for a complete list of all carbon sources used). We can represent a metabolic phenotype as a binary string, whose *i*-th entry is equal to one (Figure 1), if a network is able to sustain life when *C_i_* is the only available carbon source. A network able to sustain life in complex environments with multiple carbon sources has phenotypes in which many of these entries are equal to one.

Metabolic phenotypes, as defined here, can be computed from metabolic genotypes using flux balance analysis. Flux balance analysis is a computational tool that relies both on stoichiometric information about chemical reactions occurring in a cell, as well as on an objective function such as the production of biomass precursors. For a given nutritional environment, it computes allowable rates at which individual reactions proceed in a metabolic steady state, and these rates in turn determine whether all necessary biochemical precursors can be produced. Its qualitative predictions – growth or no growth – are in good agreement with experimental data for well-studied model systems [7],[8].

We here study the evolution of metabolic networks in the space of the genotypes just defined. Genotypes can change through the elimination of chemical reactions caused by loss of function mutations in enzyme-coding genes. Many such mutations do not abolish a network's ability to sustain life [4], [7]–[17]. Genotypes can also change through addition of chemical reactions, which occurs at appreciable rates in prokaryotes through horizontal gene transfer [9],[18]. This motivates our choice of a prokaryotic network – that of *E. coli* – as the departure point of our work [19]. Two further reasons compelled us to choose specifically the *E. coli* network. First, it is perhaps the most prominent and well-studied example of a metabolic network in a free-living organism. Second, more effort has been devoted to studying its robustness than for other networks [7], [8], [14], [16], [20]–[25]. For these reasons we also wanted to compare properties of the *E. coli* metabolic network with those of the sampled networks that our approach generates.

Mutations and horizontal transfer can sometimes affect more than one enzyme-coding gene (reaction), but we focus here on the individual reaction as the elementary unit of change. Each such change transforms a network into one of its immediate neighbors differing from it by one reaction. We refer to all of a network's neighbors as a network's neighborhood. Methodologically, our approach bears resemblance to that of an earlier study [16] which asked how minimal genomes evolve from the *E. coli* genome through metabolic gene loss. However our method is new in that we do not limit ourselves only to the elimination of chemical reactions but 1) we allow for the addition of metabolic reactions, which allows us to explore a vast genotype space, 2) our analysis is not limited to *E. coli*, and 3) we also explore a very large number of different environments.

In this context, we ask several fundamental questions about the organization of genotype space, and about the ability of metabolic networks to find evolutionary innovations in this genotype space. How different can the organization of two metabolic networks be while still preserving similar phenotypes? How many mutational steps are needed to get from a network with a given phenotype to one with a very different phenotype? How different are the new phenotypes that a network encounters in its immediate neighborhood during evolution? The answers to these questions can not only elucidate why metabolic networks are robust to mutations [4], [7], [8], [12], [26]–[30]. Even more importantly, they also tell us how metabolic innovations can arise through a metabolic network's exploration of a vast space of possible genotypes.

## Results

### Networks supporting life in one environment can have very different essential reactions

We begin our analysis with a simple phenotype, a metabolic network's ability to produce all biochemical precursors from a single carbon source, glucose, in an aerobic minimal medium (see Text S1 for a list of all environmental metabolites). The *E. coli* metabolic network [19], excluding 205 transport reactions, catalyzes 726 out of the “universe” of 5870 reactions we consider (see Text S1 for details on reaction compilations). Its immediate neighborhood in genotype space consists of the 5870 networks that differ from the *E. coli* network by one (added or eliminated) reaction. Addition of a reaction to a network would not impair its ability to grow on glucose, but elimination of a reaction might. Out of the 726 *E. coli* reactions, 210 reactions are essential and cannot be removed without abolishing growth on glucose minimal medium. Thus, only 3.6% (210/5870) of the entire neighborhood, and only 29% (210/726) of those neighbors with one deleted reaction, are not able to sustain life on glucose minimal medium.

**Figure 2:**
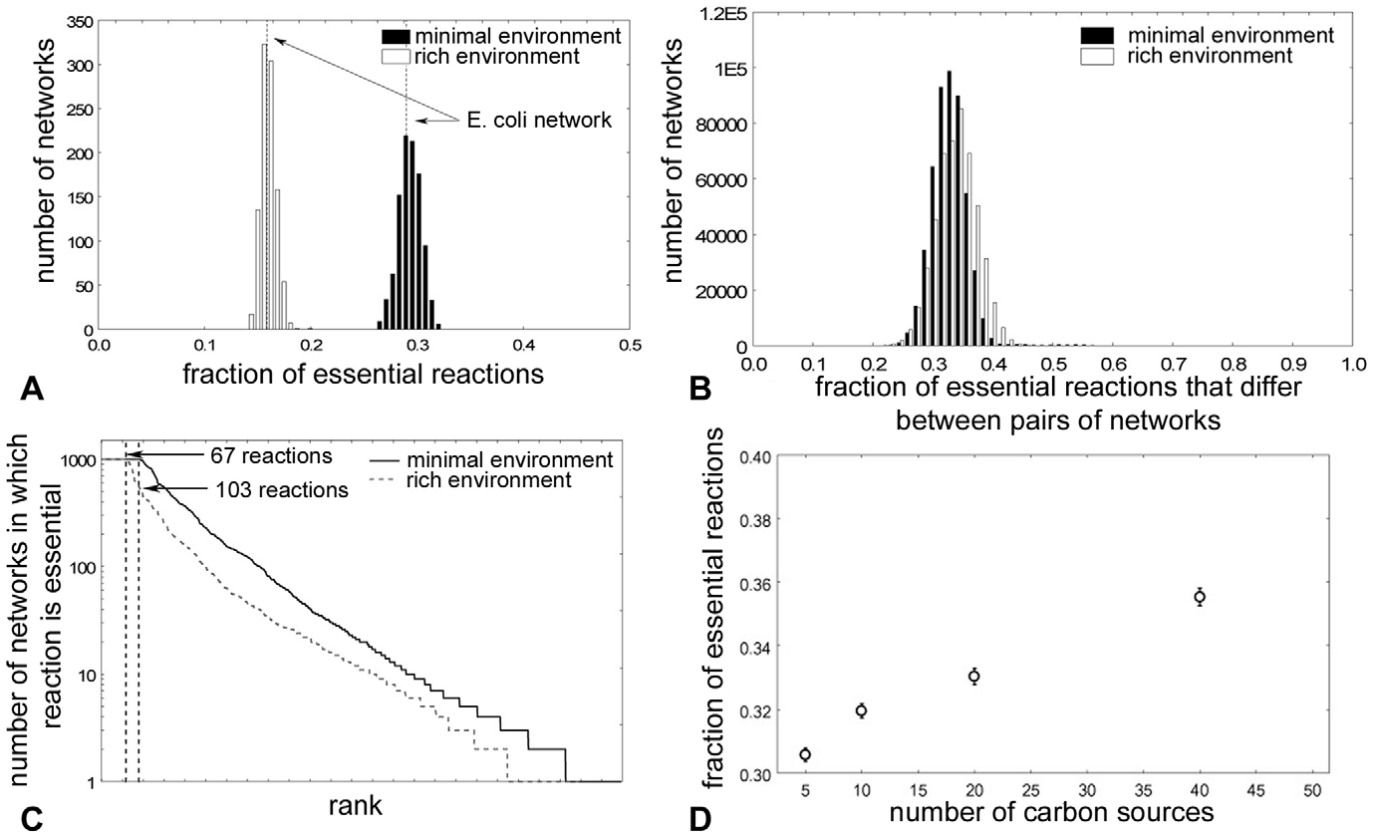
Essential reactions differ dramatically between metabolic networks with the same metabolic abilities. (A) Distribution of the fraction of essential reactions in 1000 random networks viable in minimal or rich glucose containing medium. (B) Distribution of the fraction of essential reactions shared among pairs of these 1000 random networks. (C) Rank plot of reaction essentiality. Reactions essential in all of the 1000 random viable networks are given the lowest rank of one. (D) The average fraction of essential reactions (vertical axis) as a function of the number of carbon sources a network can sustain life in (horizontal axis). Each point is an average of 100 networks (whiskers: 95% confidence interval).

Are metabolic networks that are very different from the *E. coli* network, but that can also sustain life on glucose similarly robust? To address this question, we analyzed 1000 such networks (Figure 1). These networks were the end points of 1000 long random walks of 10^4^ mutational steps each through genotype space that started from the *E.coli* network. Figure S1 shows the evolution of genotype distance and network size in one such random walk. Each step consisted of the random addition or deletion of one chemical reaction and was required to preserve the ability to sustain life on glucose minimal medium. For brevity, we will call the end-point of such a random walk a *random viable metabolic network* with a given phenotype. We emphasize that the number of reactions in the random viable metabolic networks is similar to that of the *E. coli* metabolic network (see Text S1 for algorithmic details). We examined the neighborhood of each of these 1000 random viable networks to identify essential reactions in them. Figure 2a shows the distribution of the number of essential reactions. It varies across a narrow range between a minimum of 213 (26.4%) and a maximum of 257 (32.4%) reactions. The robustness of the *E. coli* network lies in the bulk of this distribution, and is thus not atypical. This suggests that for a typical metabolic network with a given phenotype, many different mutational changes leave the network's ability to sustain life in a given environment unchanged.

How different are the networks that can sustain life in this simple environment? We addressed this question in two complementary ways. First, we asked how many essential reactions differ between each network pair drawn from the 1000 random viable networks we had generated previously. Specifically, we represented the set of all essential reactions by a binary vector. For each of the 1000 random viable networks, this vector contained a ‘1’ for a reaction that was essential in the respective network, and a ‘0’ for a reaction that was nonessential. We calculated the normalized Hamming distance between these vectors for each pair, which is the fraction of entries at which these vectors have different values. This distance ranges from zero if a network pair has completely identical essential reactions to one if a network pair has no essential reactions in common. Figure 2b shows the distribution of the fraction of essential reaction that two networks have in common. On average, 32.9% of essential reactions are different in two random viable networks with the same phenotype. If we exclude reactions from this analysis that are essential in all 1000 networks, then 74% of essential reactions differ among networks.

**Figure 3:**
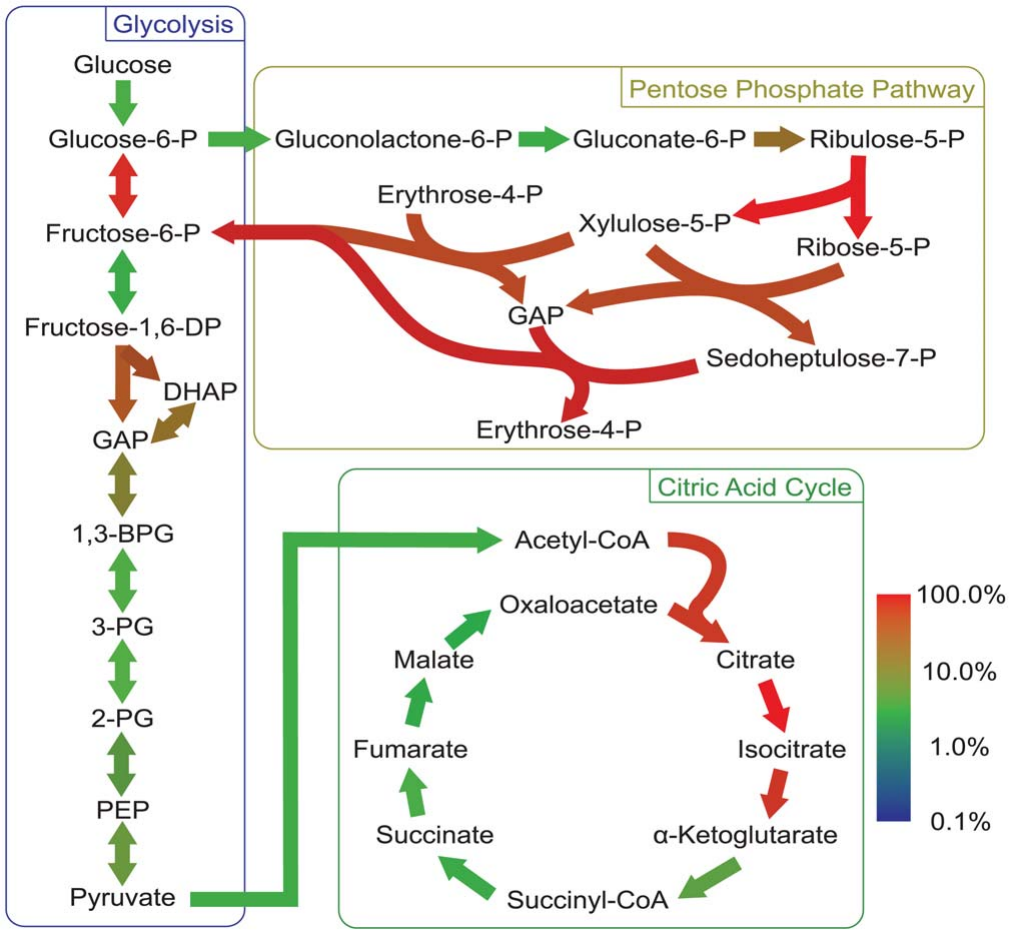
Reaction essentiality in central metabolism. Color-coded map of reactions in central energy metabolism that appear rarely (blue) or frequently (red) as essential in 1000 random viable metabolic networks. The color is in logarithmic scale indicating that most reactions even in this most central part of metabolism are essential only in a small fraction of networks with a given metabolic phenotype.

We next ranked all reactions according to the number of networks (among 1000) in which they were essential. Reactions essential in all 1000 networks received the lowest rank, and reactions that were essential in successively fewer networks received increasingly larger ranks. This ranking indirectly estimates the abundance of alternative pathways around any given reaction in a random viable metabolic network. If there are many alternative pathways, then the reaction will rarely appear as essential; if there are no alternate pathways, the reaction will appear as essential in all metabolic networks. The majority (4550) of reactions were never essential. Among the 1420 reactions that were essential in at least one network, only a small minority of 7.3% (103) reactions were essential in all networks. As an example, Figure 3 shows a measure of the reaction rank for a small subset of reactions, the key reactions in central energy metabolism (glycolysis, pentose phosphate shunt, citric acid cycle) color-coded according to whether they are rarely (blue) or frequently (red) essential. All of the 26 reactions were essential in more than one percent of the 1000 random viable networks. Around 46 percent of the reactions (12/26) were essential in more than 10 percent of the networks. Merely three reactions were essential in almost all of the networks. They come from glycolysis (glucose 6-phosphate isomerase), the citric acid cycle (aconitase), and from the pentose phosphate pathway (ribulose 5-phosphate 3-epimerase,). Note that two reactions that belong to the same (apparently unbranched) pathway of Figure 3 may show different essentiality. This can be understood by considering that for each reaction there may be a different number of alternative pathways (whose reactions are not shown in the figure) but that can compensate for the loss of the reaction.

To validate our analysis of reaction essentiality with empirical data, we tested the following prediction: If a reaction is frequently essential in our random viable metabolic networks, then its enzyme-coding genes should also occur in a large number of different genomes. This is indeed the case, as we show in Figure S4. The figure demonstrates that the frequency of a reaction as essential and the number of prokaryotic genomes carrying an enzyme-coding gene that catalyzes this reaction are positively correlated (Pearson's r = 0.45 and p = 2.2×10^−16^). For this analysis we used the information available in the KEGG database [31],[32]. Taken together, these observations show that networks with the same phenotype are highly plastic in their organization. Many essential reactions typically differ between pairs of such networks. This holds even for reactions in the most central parts of metabolism.

### Networks supporting life in one environment can have very different genotypes

**Figure 4:**
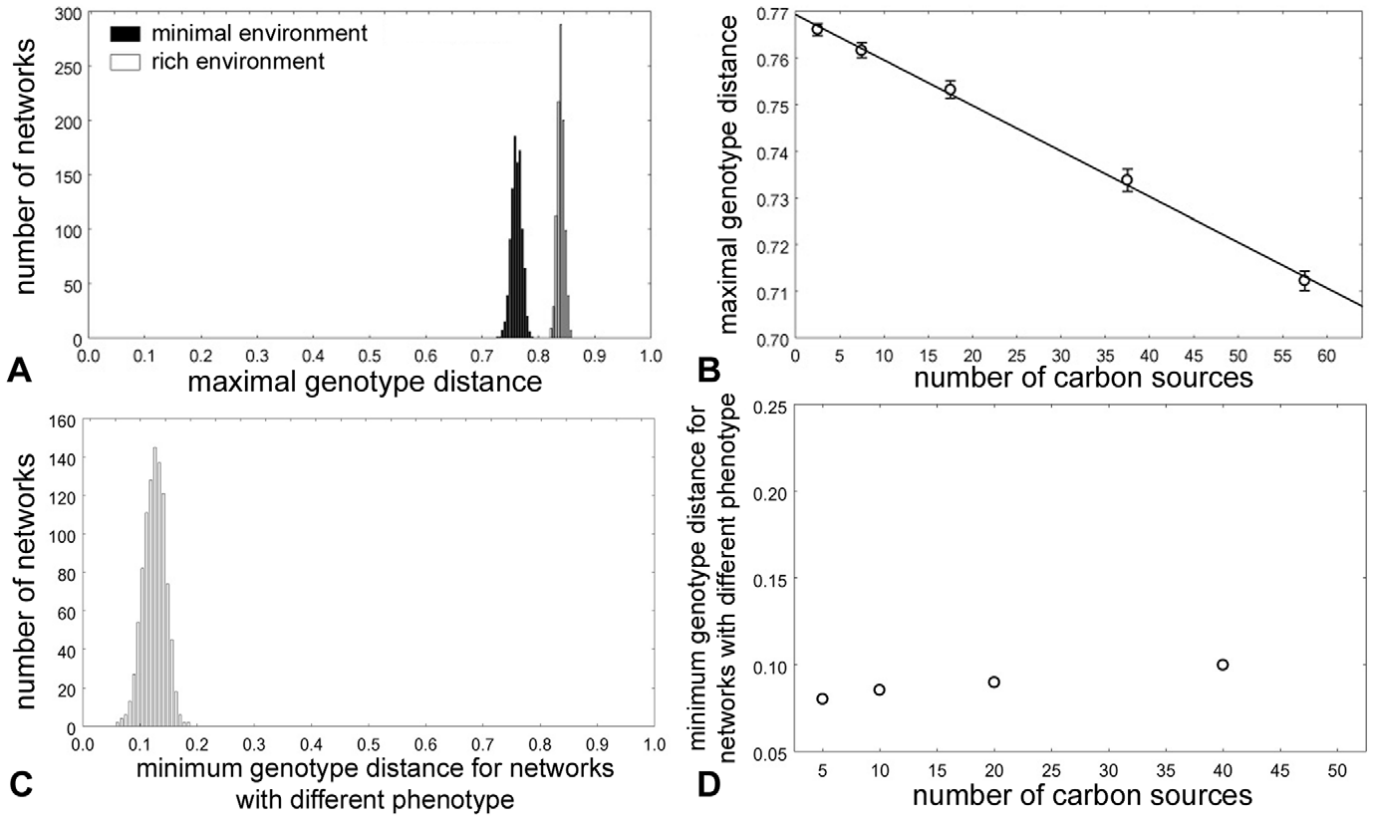
Metabolic networks with the same phenotype can have vastly different genotypes. (A) Distribution of maximum genotype distance between 1000 networks that are the end-points of random walks leading away from the initial (E. coli) network while preserving the metabolic phenotype. (B) Maximum genotype distances (vertical axis) between initial metabolic networks able to sustain life on a given number of carbon sources (horizontal axis) and 1000 final random viable metabolic networks. For each number of carbon sources 100 random walks of 10^4^ mutations were carried out starting from 10 different initial networks (whiskers: 95% confidence interval). (C) The distribution of minimal genotype distance between pairs of networks with different metabolic phenotypes required to sustain life on at least one carbon source. (D) Average minimal genotype distance (the mean of the distribution in (C) as a function of the number of carbon sources. The error bars are too short to be visible in this plot.

In a second effort to characterize the plasticity of network organization, we asked how distant from the *E. coli* network a network can maximally be and still preserve the ability to sustain life on a glucose-minimal medium. To do so, we generated 1000 networks from the *E.coli* network through a random walk similar to that described above, but where we forced each step of the random walk to increase the distance to the *E.coli* network. Figure 4a shows that more than three quarters of genotype space can be traversed without destroying the metabolic phenotype.

An environment in which metabolic networks have to synthesize every single biochemical precursor is demanding. Thus, our observations might depend strongly on the nature of this environment. However, this is not the case. We also examined a rich medium in which 36 biochemical precursors are provided for the cell (see Text S1 for details). In such a medium, 15.9% of reactions are essential on average (13.5% fewer than in minimal medium) (Figure 2a); the percentage of essential reactions that differ among two networks is very similar (33.8%; Figure 2b); the number of reactions that are essential in at least one environment is smaller (1304 vs. 1420); a smaller percentage (5.1%; 67 of 1304) of reactions are essential in all networks (Figure 2c; Table S1); and the maximal distance of networks to the *E. coli* network is on average 83.9%, even larger than in minimal medium (Figure 4a). Thus, evolution in a rich versus a minimal environments does not change our results dramatically. It is instructive to examine the reactions essential in all networks more closely. They are significantly enriched in reactions involved in tyrosine biosynthesis (P = 0.01), cell wall biosynthesis (P = 1.0×10^−10^), and membrane biogenesis (P = 2.8×10^−6^).

Taken together, the following picture emerges from these observations. Networks that have the ability to sustain life on a particular carbon source have many neighbors in genotype space with the same ability. By mutationally stepping from neighbor to neighbor (through addition and deletion of chemical reactions) network organization can change fundamentally without losing this ability. Two networks with this ability can contain very different sets of reactions, and very different essential reactions. Because networks with the ability to sustain life in a given environment are connected through their neighbors in genotype space (see Text S1 for details) this means that large fractions of genotype space can be traversed on evolutionary time scales without affecting any one metabolic ability.

### Metabolic networks with complex carbon phenotypes can also have very different organizations

We next turn to more complex phenotypes, namely the ability for a network to sustain life if any one of multiple carbon sources is provided in an otherwise minimal environment. We here focus on the 101 potential carbon sources annotated to have associated transport reactions in *E. coli*. Because the requirement to sustain life on an increasing number of carbon sources may increasingly constrain network architecture, our observations from above may not hold for such complex phenotypes. Figure 4b, however, shows that this is not the case. The figure examines the maximal genotype distance from the *E. coli* network achievable for networks with the same phenotype, as a function of the phenotype's complexity, that is, the number of carbon sources a network can sustain life on. This maximal distance declines by less than 10% for networks that can sustain life on 60 carbon sources. Thus, even if a network can sustain life in many different carbon-containing environments, its architecture is not highly constrained. The fraction of reactions that are essential does not change dramatically either (Figure 2d). Specifically, it increases modestly from a mean of 0.3 (Figure 2b) to 0.4 (Figure 2d) for networks that can sustain life on 5 and 60 different carbon sources, respectively. In this analysis, we used a very conservative definition of essentiality. For example, for networks able to sustain life on 60 different carbon sources, we call a reaction essential if it is required in at least one of the 60 minimal environments distinguished by these carbon sources. If we define reaction essentiality less conservatively, then the fraction of essential reactions actually decreases with an increasing number of carbon sources (Figure S2).

### Networks with different phenotypes can be found close together in genotype space

We next studied several properties of metabolic networks that relate to their ability to evolve new phenotypes. The first such property regards the minimum genotype distance of two metabolic networks with arbitrary, different phenotypes. If this distance is typically large, then it would be very difficult to reach any one phenotype from a network with a different phenotype through a modest number of genetic changes. To determine this distance, we first created a pair (G_1_, G_2_) of metabolic network genotypes with randomly chosen different phenotypes, as described in the Text S1. We then carried out a random walk that started from G_1_ and that approached G_2_ in genotype space, while leaving G_1_'s phenotype unchanged. When this random walk had reached a point where the genotype distance to G_2_ could no longer be reduced, we stopped and recorded the minimal distance thus obtained. We repeated this procedure for 1,000 metabolic network pairs with different phenotypes. Figure 4c shows a histogram of this minimal distance for networks that are required to sustain life on at least one carbon source. It is evident from the Figure that this distance is small relative to the distance between random viable metabolic networks with the same phenotype. It spans of the order of 10% of metabolic network size (circa 100 reactions). We note that this distance is an average over many and sometimes very different phenotypes, and also that it is merely an upper bound to the minimal distance between metabolic networks with different phenotypes. The reason is that we only minimized the distance between G_1_ and G_2_ by changing G_1_. Had we changed G_2_ as well we would have found even smaller minimal distances. Figure 4d shows how this distance depends on the number of different carbon sources a network can sustain life on. The figure shows, for phenotypes that can sustain life on increasing numbers of carbon sources (horizontal axis), the mean and standard error of the minimum distance between networks with different phenotypes. While the minimal distance increases with increasing numbers of carbon sources, this increase is small, of the order of 2% of the total genotype distance. Thus, complex constraints on metabolism do not dramatically increase the difficulty networks would encounter in evolving towards specific, novel phenotypes.

### Evolving networks encounter ever-new phenotypes in their immediate neighborhood

**Figure 5:**
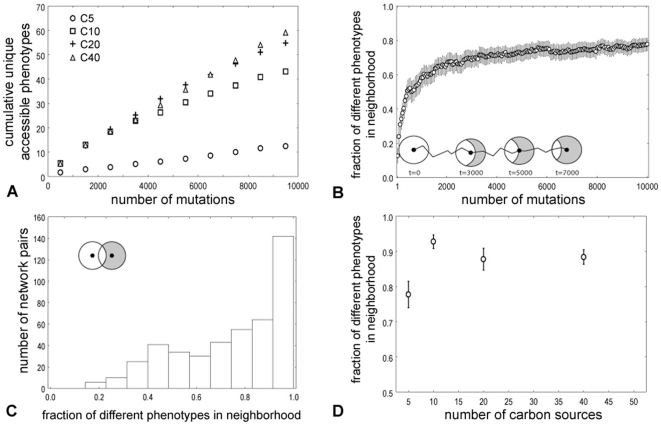
Evolving networks with conserved phenotypes can access very different novel phenotypes along their evolutionary path. (A) shows the average cumulative number of phenotypes (vertical axis) found in the neighborhood of an evolving network as a function of the number of mutations (horizontal axis) the network experienced during its evolution; (B) shows the fraction of the phenotypes in the neighborhood of the evolving network (*G_t_*) and an initial network (*G_0_*) that differ from one another. The diagram in the inset illustrates the increasing number of novel phenotypes in the evolving network's neighborhood (gray area of the circle) that are different from the phenotypes in the neighborhood of *G_0_*. For pairs of random viable metabolic networks with the same phenotype; (C) shows the distribution of the fraction of *different* phenotypes in the neighborhoods of these networks. (D) shows the mean of the distribution (C) of phenotypic differences in the neighborhood of the network pairs versus the numbers of carbon sources they can sustain growth on. Data in (A), (B), (C) and (D) are averages over 100 random walks of 10^4^ mutations starting from 10 different initial networks. In (C) only pairs of networks with the same initial network of the random walk were compared, thus 450 neighborhood comparisons. In all plots whiskers represent the 95% confidence interval.

Does the genotypic plasticity of metabolic networks facilitate the discovery of novel metabolic abilities? To address this question, we examined the novel metabolic phenotypes accessible to networks that are subject to phenotype-preserving evolutionary change. By phenotypes “accessible” to a network, we here mean all the phenotypes that can be found in the neighborhood of this network. These are novel phenotypes that can be easily reached through a single, small genetic change. Specifically, we first carried out a random walk starting from a network with a specific metabolic phenotype, and counted the cumulative unique number of phenotypes that occurred in the neighborhood of this random walker. That is, if a phenotype occurred twice, either in the neighborhood of the same network, or in the neighborhood of a network encountered previously during the random walk, we counted it only once. Figure 5a shows the cumulative number of new phenotypes that such an evolving network encounters. This number does not saturate and continues to increase even though the random walk shown here comprises many thousand mutations.

Second, we compared the phenotypes in the neighborhood of (i) an evolving network *G_t_* with unchanging phenotype, and (ii) its ancestor *G_0_* as a function of the number of mutations *t* between the two networks. Specifically, we asked for the fraction of phenotypes that *differ* between the one-neighborhoods of the two neighborhoods. If this fraction were close to one for large *t*, then even two dissimilar networks might only have access to very similar metabolic phenotypes. Figure 5b shows, as a function of *t*, the fraction of different phenotypes in the neighborhood of *G_0_* and *G_t_*. It is evident that this fraction approaches a large value very quickly, that is, even similar genotypes have access to a diverse spectrum of phenotypes.

Third, we examined the neighborhoods of multiple end points (orange circle in Figure 1) of long phenotype-preserving random walks starting from the same network. Doing so tells us how different the phenotypes accessible from very different (essentially random) metabolic networks with the same phenotype are. Figure 5c shows the distribution of this fraction of accessible but different phenotypes for 4950 network pairs. Importantly, the vast majority of phenotypes differ among these pairs. That is, phenotypes found near one network are usually different from phenotypes near another network with the same phenotype. In sum, three independent lines of evidence show that the metabolic phenotypes accessible to networks with the same phenotype differ dramatically even for moderately different networks.

Finally, we also examined how the accessibility of novel phenotypes depends on the phenotypic complexity of the evolving networks themselves, that is, on the number of carbon sources that they can support life on. In principle, all 2^101^ phenotypes are accessible from any metabolic genotype through a single mutation, regardless of the number of carbon sources the genotype is viable in (see Text S1 for a detailed explanation). However, Figure 5a and Figure S3 show that networks able to sustain life on more carbon sources encounter more novel phenotypes along their evolutionary trajectory. In addition, Figure 5d shows that the fraction of metabolic phenotypes that differ between the neighborhoods of random viable network pairs with the same phenotype is consistently large and shows no simple dependency on the number of carbon sources.

## Discussion

Metabolic networks can evolve through the elimination of individual reactions by mutation, and through the addition of new reactions by horizontal gene transfer. We here explored a vast space of metabolic network genotypes through random changes of individual reactions that preserve a network's metabolic abilities. The ability of flux balance analysis to determine metabolic phenotypes –a network's ability to sustain life in a well-defined environment containing specific carbon sources – allowed us to characterize the relationship between metabolic genotypes and phenotypes. We find that metabolic networks with the same phenotype show enormous genetic plasticity, and that this plasticity aids in the evolution of novel metabolic abilities.

Multiple experimental and computational studies show that a large fraction of enzyme-coding genes are dispensable in genome-scale metabolic networks. These networks continue to sustain life even upon removal of many apparently central and important reactions [7], [8], [12], [14], [16], [27], [33]–[36]. These studies raise the question whether such robustness is an evolutionary adaptation, evolved in response to ongoing mutational pressure. Our approach of creating multiple, essentially random viable metabolic networks with pre-defined phenotypes suggests an answer to this question for the *E. coli* metabolic network. In both a glucose-minimal and a rich environment, the fraction of reactions dispensable in the *E.coli* network is not dramatically different from that of 1,000 metabolic networks with the same metabolic phenotypes. This argues that the high robustness to gene deletions of *E. coli* metabolism may not be an evolutionary adaptation, but is rather typical of metabolic networks of comparable size. A caveat to this observation is that our approach allows modest fluctuations in reaction numbers (by about 14 percent) to facilitate the sampling of metabolic genotype space. These fluctuations may influence estimates of robustness by approximately the same amount. We will leave exploration of this influence to future work.

Our observations go beyond preceding work which showed that a reaction's essentiality may depend on the environment [28],[37]. We demonstrate that the plasticity of metabolic networks is so great that even in a single environment, different networks with the same phenotypes may show very different essential reactions. For example, only 7.3% of all reactions essential in at least one of 1,000 networks are essential in all networks. Excluding these reactions, two networks with the same phenotype differ in 74% of their essential reactions. Even in pathways as important as central energy metabolism, the vast majority of reactions are essential in only 1% of networks. One might think that networks able to thrive on many different carbon sources might show vastly more essential reactions. However, this is not the case. Reaction essentiality depends only modestly on the number of carbon sources a network can sustain life on.

Gene essentiality thus strongly depends on a network's genotype, which is highly malleable. Even organisms with similar metabolic abilities may thus show very different dispensable genes in a given environment. These observations have implications for the design of antimetabolic drugs that inhibit specific metabolic reactions. Specifically, an evolutionary approach like ours may be highly useful in identifying reactions that are essential in most networks with a given metabolic phenotype, as a precursor to rationally designing drugs inhibiting these reactions. The more frequently essential a reaction is, the smaller the likelihood that a cell can circumvent it through addition or deletion [27] of other reactions. For example, the major antimetabolic antibiotics – sulfonamides and trimethoprim – inhibit two different reactions (dihydropteroate synthetase and dihydrofolate reductase) leading to tetrahydrofolate, an essential precursor for nucleic acid synthesis. These two reactions, however, are essential in only 40 percent of networks able to sustain life in rich medium. Figure S5 shows some of the ways by which nonessentiality arises in this case. Multiple bacterial species, for example, bypass the need for dihydrofolate reductase in the synthesis of nucleotide precursors, using a flavin-dependent thymidilate synthase instead [38]. A better target in the same pathway would be the enzyme dihydrofolate synthase, which our approach finds to be essential in all networks (Figure S5) In a similar vein, it is no coincidence that a broad class of antibiotics (penicillins, bacitracin, cephalosporins, carbapenems, vancomycin etc.) target synthesis of cell walls and membranes: Among the reactions found to be essential in all networks (Table S1), cell wall and membrane biosynthesis reactions are highly enriched. Thus, our approach lends itself to a pre-screening of metabolic reactions or reaction classes for drug targeting.

Our analysis shows that vastly different networks with the same phenotype can be connected through paths of single mutations (reactions additions/deletions) in genotype space. Specifically, these paths can traverse more than three quarters of genotype space without destroying a given phenotype. This phenomenon does not depend strongly on the evolutionary constraints on a metabolic network, that is, on the number of carbon sources a network is required to sustain life on. These observations are reminiscent of genotype networks or neutral networks that have been characterized for RNA, protein, and transcriptional regulation circuits [39]–[44]. In these networks, genotypes with the same phenotype form large sets in genotype space, sets that can be connected through many single, small mutational changes. For example, proteins with the same tertiary structure and function (phenotype) often share a common ancestor, but their amino acid sequences (genotypes) have diverged beyond recognition [45],[46]. The existence of such genotype networks – and the robustness it implies – facilitates the evolution of new molecular functions [47]–[50].

We here provide two lines of evidence that genotype networks may also facilitate the evolution of new metabolic phenotypes, the ability to survive on previously not utilizable carbon sources. First, we show that networks with different and arbitrary phenotypes can be found close together in genotype space. This means that from any one network, only a small fraction of genotype space needs to be traversed to find any given, novel phenotype. Second, we also analyze the neighborhood of different neutral networks with the same phenotype. This neighborhood consists of all networks that differ in only one reaction from a focal network. They are thus accessible from this network through a single mutation. We find that the neighborhoods of different networks contain very different novel phenotypes. This means that by traversing a large fraction of genotype space without changing the phenotype, one can render different novel phenotypes accessible (Figure S6). Put differently, even microorganisms with identical phenotypes may be able to access very different novel phenotypes. This observation points to the need to carefully choose organismal strains for engineering of novel metabolic abilities, such as the production of biofuels, or the degradation of toxic compounds in bioremediation. The right choice may mean that only a small alteration, such as the addition of one reaction to a metabolic network, is sufficient to produce a desired new phenotype. Consider the example of the carbon source melibiose, a sugar similar to lactose and made of the same two monosaccharides (galactose and glucose) but differing in the glycosidic link between them. While lactose can be metabolized by many microbes, melibiose is a less commonly utilizable compound. The metabolization also requires different enzymes (α-galactosidase for melibiose and β-galactosidase for lactose). The metabolic ability to use melibiose is desirable, for example in yeast, where cells have been engineered to utilize melibiose to improve efficiency and reduce waste in fermented dairy products [51]. Among the networks with identical metabolic phenotypes that we examine, there are networks where adding the α-galactosidase reaction is sufficient to endow the network with melibiose utilization. In contrast, in other networks with the same phenotype the addition of this reaction is not sufficient (even though both networks are able to grow on glucose). The reason is that these latter networks are unable to excrete the excess galactose from the degradation of melibiose. Another example involves the addition to a network of a single reaction catalyzing the transfer of a phosphor group from a phospho-histidine to galactitol. This reaction produces galactitol 1-phosphate, and it enables the network to grow on galactitol. In another network with the same phenotype, the addition of this reaction does not have the same result. The reason is that the first network contains other reactions that enable it to convert of galactitol 1-phosphate into galactose, which it can grow on.

We next motivate the choice of metabolic network sizes for our work. Flux balance analysis has been used to show that a significant number of reactions in *E. coli*, when removed, show no impact on optimal growth in several different environments [52]. This observation might lead one to suppose that phenotype-preserving paths through genotype space are long merely because many reactions are never essential. However, this is not the case. For example, although the fraction of essential reactions in *E. coli* is merely 28% when considering a glucose minimal environment, this fraction rises to 43% when considering growth on each of the more than 81 carbon sources we examined here. In addition, when considering the influence of the genetic background, we observe that 66% of the reactions appear as essential in at least one of the many randomized viable metabolic network in a glucose minimal environment, and 81% of reactions become essential when we consider the full spectrum of 81 carbon sources. This fraction of essential reactions would undoubtedly have risen further if we had the computational means to analyze additional carbon sources and genetic backgrounds. Taken together, these observations mean that essentiality of reactions depends on environment and genetic background, and that there may not be a meaningful reduced reaction set that is always under selection. These observations, and our desire to compare properties of our sampled networks to the *E. coli* network prompted our choice of network size.

Flux balance analysis has limitations in how precisely it can predict growth or by-product secretion after gene knockouts [5], which may depend on the choice of optimization principle [53] and flux maximization method [7]. These limitations are connected to how metabolic genes are regulated, and they do not affect our study because we are not concerned with regulatory evolution. For our purposes, it is sufficient to evaluate if an organism represented by a metabolic network is viable in principle, based on the complement of enzymes it carries and the biomass precursors it can synthesize given a spectrum of nutrients.

The potential problem of limited and likely biased information about the set of biochemical reactions that occur in nature does not affect our results qualitatively. The reason is that any increase in the number of known biochemical reactions will cause the appearance of alternative pathways, lowering the number of essential reactions, and thus increasing the robustness and the plasticity of metabolic networks.

Aside from these caveats, the biggest limitation of the approach presented here lies in its computational demands. Determining the metabolic phenotypes of networks in the neighborhood of a single genome-scale network for 101 carbon sources requires the solution of 5.85×10^5^ ( = 101×5800) complex linear programming problems [5]. For our simulations we analyzed more than 20’000 such genomes and this was currently at the limit of computational feasibility. This limitation will undoubtedly be ameliorated with time.

In sum, the approach proposed here can provide various insights into the organization of metabolic networks. It demonstrates that the architecture of such networks shows high plasticity, even for single environments, a property that facilitates the evolution of new metabolic functions. It suggests a method to target metabolic reactions for rational drug design, and shows that the plasticity of metabolic networks creates both opportunities and constraints for the evolution of novel metabolic abilities.

## Methods

### Random walks in genotype space

We explore the vast space of metabolic networks by long random walks that leave a network's ability to synthesize all essential biomass components unchanged. Each step of the random walks we use has two parts. The first part consists of mutation, the deletion of a randomly chosen reaction from a network, or the addition of a new randomly chosen reaction from the global reaction set above. We constrain variation in the number of reactions in this random walk by means of a bias in the choice of mutation that depends linearly on the number of reactions in the metabolic network (see Text S1). With this procedure, the networks have always approximately 1000 reactions throughout the simulations. In the second part of a random walk's step, we apply flux balance analysis to verify that the new metabolic network still has the same phenotype, i.e., that it can still grow on the same specific set of carbon sources. If so, the mutated network is accepted and the next step of the walk starts with the mutated network; if not, the mutated network is rejected, and the next step of the random walk starts with the previous (unmutated) network.

Methods are described in greater detail in the Text S1.

## Supporting Information

Text S1 Detailed description of simulation conditions and methods
(0.14 MB PDF)

Figure S1 Random walks in genotype space. a) Autocorrelation function of growth flux in an unbiased random walk of 10'000 generations starting from the E. coli metabolic network. The autocorrelation function was calculated for the last 5'000 generations. b) A sample trajectory of a random walk starting from the E.coli metabolic network, showing both the number of reactions in the evolving network, as well as the genotype distance (normalized Hamming distance) between the evolving network and the initial network. When the genotypes of both networks are represented by binary vectors indicating the presence or absence of reactions (see Figure 1a), the normalized Hamming distance corresponds to the fraction of entries in these two vectors that are different.
(0.07 MB TIF)

Figure S2 The fraction of reactions essential in a complex environment decreases with environmental complexity. Average fraction of essential reactions (vertical axis) as a function of the number of carbon sources a network can sustain life in (horizontal axis). A reaction is called essential here, if it is essential in an environment that contains all of the carbon sources a network is required to grow on. For each number of carbon sources 10 different initial networks were generated, as described in Methods, and for each of these 10 networks 10 random walks were carried out. Each circle on the plot is thus based on 100 networks (whiskers: 95% confidence interval). See Methods for details on how the initial networks were generated.
(0.06 MB TIF)

Figure S3 Networks that can grow on more carbon sources encounter more novel phenotype during their evolution. The average cumulative number of phenotypes (vertical axis) found in the neighborhood of an evolving metabolic network at the endpoints of 100 phenotype-preserving random walks is shown as a function of the number of carbon sources the initial networks can grow on. For each number of carbon sources shown, the data is an average over 10 independently generated initial networks, and over 10 random walks starting from each of these 10 networks.
(0.06 MB TIF)

Figure S4 Reaction essentiality and gene appearance in prokaryotic genomes. Correlation of frequency of reaction essentiality in random metabolic networks and number of genomes carrying an enzyme-coding gene catalyzing that reaction. Pearson's r = 0.45; p = 2.2×10−16. This analysis uses enzyme-coding genes from 875 prokaryotic genomes in the KEGG database
(0.10 MB TIF)

Figure S5 Reactions in tetrahydrofolate biosynthesis and their essentiality. We found that the reaction dihydropteroate synthetase, a target of sulfonamides, is essential in 41% of the metabolic networks we studied, while the other reaction producing dihydropteroate is essential in 56.1% of networks. In the remaining 2.9% of networks, both reactions appear, but none are essential. These observations have a straightforward explanation. Dihydropteroate is an essential metabolite. Because only two alternative reactions exist to make dihydropteroate, whenever one of these reactions is missing, the other is an essential reaction. Whenever both reactions are present, neither reaction is essential. For the production of tetrahydrofolate from dihydrofolate, there exist, similarly, two parallel dihydrofolate reductase reactions. These reactions are the target of trimethoprim. The reactions are only distinguished by the molecule that acts as the electron donor, either NADH or NADPH. Individually, these reactions appear as essential in only 30%–40% of networks. In addition, only 66.2% of networks cannot tolerate the removal of both reactions. The reason is that there are alternative paths (not shown) that bypass the direct production of tetrahydrofolate from dihydrofolate.
(0.06 MB TIF)

Figure S6 The connectedness of metabolic networks with the same phenotype facilitates access to new metabolic phenotypes. The rectangle symbolizes genotype space, and the grey circles symbolize metabolic networks with a given metabolic phenotype. The colored circles stand for metabolic networks with a novel phenotype. Different novel phenotypes (different colors) are accessible from different networks (points) in genotype space with the same phenotype.
(0.08 MB TIF)

Table S1 List of reactions that appear frequently as essential in random metabolic networks
(0.43 MB XLS)
