# Supplementary material for: Evolutionary Plasticity and Innovations in Complex Metabolic Reaction Networks
Source: PLoS Comput Biol. 2009 Dec 18;5(12):e1000613. doi: 10.1371/journal.pcbi.1000613 (PMC2785887; doi:10.1371/journal.pcbi.1000613)
Supplement: Text S1 — Detailed description of simulation conditions and methods (0.14 MB PDF) [file pcbi.1000613.s001.pdf]

Supporting Information

Flux balance analysis

Flux balance analysis (FBA) is a computational approach to characterize the behavior of large (>10^3 reactions) chemical reaction networks [3,5,44-46]. In FBA, a network is represented by a set of stoichiometric equations describing chemical reactions. FBA takes advantage of the invariance of metabolite concentrations in a metabolic network that is in steady-state. This invariance implies that only some distributions of metabolic fluxes – rates at which individual reactions proceed – do not violate the law of mass conservation. Among these *allowed* steady-state fluxes, FBA can identify fluxes that have particular properties of interest in a given environment, defined by a maximum influx of external nutrients. We are here interested in one key property, namely whether a given metabolic network can sustain life in a given environment. That is, can it produce all key biochemical precursors necessary to sustain growth and energy production? Flux balance analysis allows us to answer this question. In our work we use a set of biochemical precursors from *E. coli* [47-49] as the set of required compounds a network needs to synthesize, by using linear programming to optimize the flux through a specific objective function, in this case the reaction representing the production of biomass precursors we are able to know if a specific metabolic network is able to synthesize the precursors or not. The precursors include all 20 proteinaceous amino acids, nucleotides, deoxynucleotides, putrescine, spermidine, 5-methyltetrahydrofolate, coenzyme-A, acetyl-CoA, succinyl-CoA, cardiolipin, FAD, NAD, NADH, NADP, NADPH, glycogen, lipopolysaccharide, phosphatidylethanolamine, peptidoglycan, phosphatidylglycerol, phosphatidylserine and UDPglucose. Flux balance analysis relies on linear programming [50] to identify network properties of interest. We here used the packages CPLEX (11.0, ILOG; http://www.ilog.com/) and CLP (1.4, Coin-OR; https://projects.coin-or.org/Clp) to solve the associated linear programming problems.

We studied metabolic networks in one main aerobic environment, a minimal environment composed of one or more carbon sources, oxygen, ammonia, inorganic phosphate, sulfate, sodium, potassium, iron, protons and water. When studying different growth phenotypes of a particular metabolic network we here focus on carbon sources, and thus vary only the carbon source in this minimal aerobic environment. For example, when we say that a network is able to sustain life on five specific carbon sources, we mean that it produces all essential biosynthetic precursors (a non-zero growth flux) when each of these carbon sources is provided as the sole carbon source in a minimal medium. This implies of course that any subset or combination of these five carbon sources would also suffice to sustain life. The 101 possible carbon sources we study here represent a tiny fraction of 101/550=18.3% of all carbon-containing metabolites in *E. coli*, and an even smaller fraction 101/4425=2.2% of carbon containing metabolites in our "universe" of metabolites (Figure S1a). Many metabolites other than those from *E. coli* can and do serve as carbon sources for other prokaryotes. Computational limitations prevented us from analyzing more complex carbon phenotypes.

For some analyses, we also used a rich aerobic environment [51]. This environment is composed of 36 metabolites, which includes the proteinaceous aminoacids , carbon dioxide, thiamin, nicotinamide mononucleotide, pantoate, and all the metabolites available in the minimal environment.

The global reaction set

Each metabolic network is a point in a much larger genotype space of networks. For the "universe" of reactions that can occur in these networks we used data from the LIGAND database [52] of the Kyoto Encyclopedia of Genes and Genomes (KEGG; http://www.genome.ad.jp/kegg/ligand.html) [53]. The LIGAND database is a database of chemical compounds and reactions in biological pathways that is compiled from

pathway maps of metabolism of carbohydrates, energy, lipids, nucleotides, amino acids and others. Also included in the database is the list of recommended names for enzymes given by the Nomenclature Committee of the International Union of Biochemistry and Molecular Biology (NC-IUBMB) (http://www.chem.qmul.ac.uk/iubmb/enzyme/) which includes all categorized enzymes (oxidoreductases, transferases, hydrolases, lyases, isomerases and ligases).

We used specifically the REACTION and COMPOUND sections of the LIGAND database to construct our global reaction set. From this data we pruned (i) all reactions involving general polymer metabolites of unspecified numbers of monomer units ($C_2H_6(CH_2)_n$), or, similarly, general polymerization reactions that were of the form A_n+ B → A_{n+1}, because their abstract form makes them unsuitable for stoichiometric analysis, (ii) reactions involving glycans, because of their complex structure, (iii) reactions that were not stoichiometrically or elementally balanced, and (v) reactions involving complex metabolites without chemical information about their structure.

The starting point of our work is the *E. coli* metabolic network (IJR904) [19] which comprises 726 reactions (excluding transport reactions). We merged all reactions in the *E.coli* network with the reactions in the KEGG dataset. (Only few *E.coli* reactions, such as specific nutrient or waste transport reactions necessary for FBA, and some specific polymerization reactions were not already in the KEGG database.) After these steps of pruning and merging, our global reaction set consisted of 5870 reactions and 4634 metabolites.

The set of networks able to sustain life on a given set of carbon sources is connected.

We note that two network genotypes able to sustain life on a given set of carbon sources can be reached from one another through single mutations in genotype space without

abolishing this ability. To see this, consider the set of reactions R_1 and R_2 that occur in two arbitrary such networks. Denote the network formed of the union of these reaction sets as $R_1 \cup R_2$. Note that the addition of a chemical reaction to any network will not abolish its ability to sustain life on any given spectrum of carbon sources. This means that there exists a sequence of single reaction changes $(\mu_1,\ldots,\mu_n)$ that leads from R_1 to $R_1 \cup R_2$, as well as another sequence $(\upsilon_1,\ldots,\upsilon_m)$ that leads from R_2 to $R_1 \cup R_2$. Denote for any mutational change υ its opposite as $\overline{\upsilon}$. That is, if υ is the deletion of a reaction *r*, then $\overline{\upsilon}$ is the addition of the same reaction to a network that does not contain it, and vice versa. It follows from the above considerations that the sequence of mutations $(\mu_1,\ldots,\mu_n,\overline{\upsilon}_m,\ldots,\overline{\upsilon}_1)$ lead from R_1 to R_2 without abolishing the ability to sustain life.

Random walks in genotype space

We explore the vast space of metabolic networks by long random walks that leave a network's ability to synthesize all essential biomass components unchanged. In each step of such a walk, one reaction is eliminated or added to a network. During a sufficiently long random walk, the reactions in a network become effectively randomized, yet the phenotype remains constant. We are well aware that recombination through unequal cross-over or horizontal gene transfer may change more than one reaction at a time, but we focus here on individual reactions, because they are the smallest sensible unit of change. In biological evolution, natural selection probably plays a major role in changing the structure of biological networks. For example, the addition of a reaction to a metabolic network may become favorable in a new environment, and go to fixation without affecting the network's ability to sustain life in the original environment. Because the detailed modeling of these and similar evolutionary dynamics would require us to make many *ad hoc* assumptions, we instead focus on the more tractable question whether changes can preserve metabolic phenotypes.

Each step of the random walks we use has two parts. The first part consists of mutation, the deletion of a randomly chosen reaction from a network, or the addition of a new randomly chosen reaction from the global reaction set above. We constrain variation in the number of reactions in this random walk by means of a bias in the choice of mutation that depends linearly on the number of reactions in the metabolic network. Specifically, the probability that a reaction is deleted (as opposed to added) is given by $p_{del}=R/R_0-0.5$, where R is the number of reactions in the current network, and R_0 is the number of reactions in the initial network, i.e., at the start of the random walk. With this procedure the networks have approximately 1000 reactions throughout our random walks, because we used the E. coli network as the starting network for these random walks. Without constraint, the number of reactions in a metabolic network would steadily increase, because networks with more reactions are more likely to sustain life in a given environment. We note that our approach allows an increase in the number of reactions of roughly 14 percent relative to the starting (*E.coli*) network. It would thus not bias our estimates of the robustness of randomized viable networks by more than that amount. In the second part of a random walk's step, we apply flux balance analysis to verify that the new metabolic network still has the same phenotype, i.e., that it can still grow on a specific set of carbon sources. If so, the mutated network is accepted and the next step of the walk starts with the mutated network; if not, the mutated network is rejected, and the next step of the random walk starts with the previous (unmutated) network.

In carrying out these random walks, it is important to proceed for as many steps as are needed to "erase" the "memory" of the initial state. To arrive at a heuristic criterion for the required number of steps, we determined, first, the autocorrelation function of the growth flux [5,19] along a random walk. This autocorrelation function decays to a value of zero in around 500 (Figure S1a) mutational steps. Unless otherwise mentioned, the number of mutational steps we use in our analysis is 10^4, and thus vastly exceeds

this required number of steps. Second, we recorded the (Hamming) distances of the random walker to the initial network during random walks. This distance first increases, and then reaches a stochastic equilibrium after about 5,000 steps, a number smaller than the 10^4 steps we routinely used. Finally, we note that the networks we studied have less than 10^3 reactions. In a random walk of 10^4 steps, each reaction is thus mutated many times over. Taken together, these observations show that 10^4 steps are more than sufficient to effectively randomize the initial network. We will refer to the end-point of such a random walk as a *random viable metabolic network* with a given phenotype. It may be very different from a random sample of chemical reactions from the whole set of reactions we consider (Figure S1a), which may not sustain life in any environment.

We call the random walk defined above an *unbiased* random walk, because it does not lead into a particular direction. To study different aspects of network evolution, we also use several random walks with the following specific biases.

First, to study the diameter of the set of genotypes with a given phenotype, it is necessary to obtain metabolic networks whose Hamming distance to the starting network is as large as possible. To this end, we used a *forced* random walk. Here, whenever a reaction that occurred in the initial network is removed from the network, we do not allow it to be added again. In this manner, no individual step of the walk can decrease the Hamming distance to an initial network.

Second, to obtain networks that grow on a specific target number k_T of carbon sources (without regard to the identity of these carbon sources), we start with a network that sustains growth on some number k_0 of carbon sources. If $k_0 > k_T$, we allow only mutations that maintain or decrease the number of carbon sources a network is able to grow on. Specifically, we revert a newly mutated network to its previous state whenever the number of carbon sources that it grows on is greater than the previous state, or

smaller than the target number of carbon sources. If $k_0 < k_T$, we allow only mutations that maintain or increase the number of carbon sources a network is able to grow on.

Third, to find a network that grows on a specific target set of carbon sources (C_0, C_1, …, C_k), i.e., a network whose phenotype P_T is a specific binary vector (Figure S1), we accept new mutations only when they decrease or do not alter the Hamming distance between the current phenotype P and the target phenotype P_T , $d(P,P_T)$.

Characterizing maximum genotype distances

To study the maximal distances of two genotypes with the same phenotype, we began with the *E. coli* network, and first obtained one network expressing different phenotypes distinguished by the number k=5,10,20,40 of carbon sources they grow on. From each of these initial networks, we performed 100 forced random walks of 10^4 mutational steps each that conserved the phenotype of the initial network. We then recorded the distribution of the Hamming distances between the genotype G_0 of each starter network and the maximally distant network G_T at the end of the random walk, and studied the properties of this distribution as a function of k.

Characterizing minimum genotypic distances for networks with different phenotypes

To characterize the minimal genotype distance that separates a pair of genotypes (G_1, G_2) with different phenotypes (P_1,P_2), we performed the following analysis. For each class of phenotypes that grow on k=5,10,20,40 carbon sources, we generated 100 pairs of random viable metabolic networks. Each network in a pair has a different (random) phenotype, with the constraint that both networks from a pair can sustain life on the same number of carbon sources. For each pair, we then performed a forced random walk of 1,000 steps, beginning with the first network G_1 (leaving the genotype G_2 of the

second network unchanged). Each mutation in this random walk was required to (i) keep the network's phenotype unchanged, and (ii) not increase the Hamming distance to G_1. We recorded the minimal distance encountered in this random walk.

Phenotype accessibility is independent of the number of carbon sources the metabolic network is viable in

In our simulations we always considered phenotypes based on the full spectrum of 101 carbon sources. When we perform a random walk for a network that grows on 20 carbon sources, we only allow mutations to be accepted if they leave unchanged the phenotype (neither introduce the ability to be viable in an additional carbon source, or lose the viability in one of the original 20 carbon sources). However when we check the diversity of the phenotypic neighborhoods we do not limit new phenotypes to 20 carbon sources. Instead, we consider all the 2^{101} phenotypes that 101 carbon sources allow. A network that is viable in one carbon source may have a mutant that through a reaction addition will have a phenotype viable in, for example, 20 carbon sources. In the same manner, a network viable in 20 carbon sources may, through a reaction deletion, become viable in only one carbon source. This merely serves to show that all 2^101 phenotypes are accessible in principle in all our analyses. In other words, a larger number of carbon sources does not enable more phenotypes

References

44 Edwards, J. S., Ramakrishna, R. & Palsson, B. O., Biotechnology and Bioengineering 77 (1), 27 (2001).

45 Almaas, E., Kovács, B., Vicsek, T., Oltval, Z. N. & Barabási, A.-L., Nature (427), 839 (2004).

46 Joyce, A. R. et al., Journal of Bacteriology 188 (23), 8259 (2006).

47 Pramanik, J. & Keasling, J. D., Biotechnology and Bioengineering (56), 398 (1997).

48 Neidhardt, F. C., Ingraham, J. L. & Schaechter, M., Physiology of the bacterial cell. (Sinauer Associates, Inc., Sunderland, MA, 1990).

49 Ingraham, J. L., Maalce, O. & Neidhardt, F. C., Growth of the bacterial cell. (Sinauer Associates, Inc., Sunderland, MA, 1983).

50 Murty, K. G., Linear Programming. (John Wiley & Sons, New York, 1983).

51 Wunderlich, Z. & Mirny, L. A., Biophys. J. 91 (6), 2304 (2006).

52 Goto, S., Nishioka, T. & Kanehisa, M., Bioinformatics (14), 591 (1998).

53 Kanehisa, M. & Goto, S., Nucleic Acids Research (28), 27 (2000).
