# Supplementary figures and images for: Evolutionary Plasticity and Innovations in Complex Metabolic Reaction Networks
Source: PLoS Comput Biol. 2009 Dec 18;5(12):e1000613. doi: 10.1371/journal.pcbi.1000613 (PMC2785887; doi:10.1371/journal.pcbi.1000613)

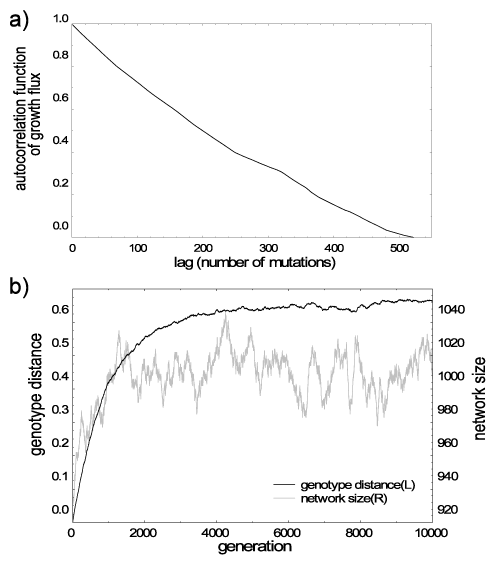

Supplement: Figure S1 — Random walks in genotype space. a) Autocorrelation function of growth flux in an unbiased random walk of 10'000 generations starting from the E. coli metabolic network. The autocorrelation function was calculated for the last 5'000 generations. b) A sample trajectory of a random walk starting from the E.coli metabolic network, showing both the number of reactions in the evolving network, as well as the genotype distance (normalized Hamming distance) between the evolving network and the initial network. When the genotypes of both networks are represented by binary vectors indicating the presence or absence of reactions (see Figure 1a), the normalized Hamming distance corresponds to the fraction of entries in these two vectors that are different. (0.07 MB TIF) [file pcbi.1000613.s002.tif]

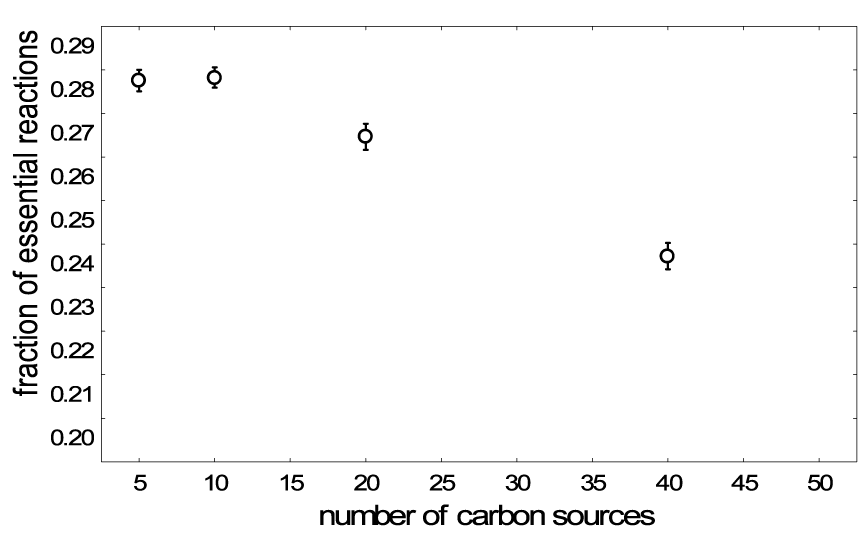

Supplement: Figure S2 — The fraction of reactions essential in a complex environment decreases with environmental complexity. Average fraction of essential reactions (vertical axis) as a function of the number of carbon sources a network can sustain life in (horizontal axis). A reaction is called essential here, if it is essential in an environment that contains all of the carbon sources a network is required to grow on. For each number of carbon sources 10 different initial networks were generated, as described in Methods, and for each of these 10 networks 10 random walks were carried out. Each circle on the plot is thus based on 100 networks (whiskers: 95% confidence interval). See Methods for details on how the initial networks were generated. (0.06 MB TIF) [file pcbi.1000613.s003.tif]

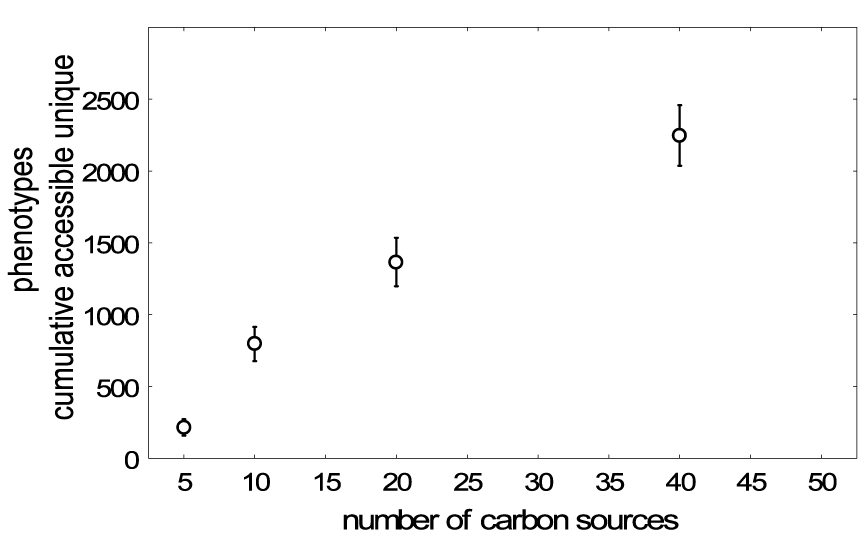

Supplement: Figure S3 — Networks that can grow on more carbon sources encounter more novel phenotype during their evolution. The average cumulative number of phenotypes (vertical axis) found in the neighborhood of an evolving metabolic network at the endpoints of 100 phenotype-preserving random walks is shown as a function of the number of carbon sources the initial networks can grow on. For each number of carbon sources shown, the data is an average over 10 independently generated initial networks, and over 10 random walks starting from each of these 10 networks. (0.06 MB TIF) [file pcbi.1000613.s004.tif]

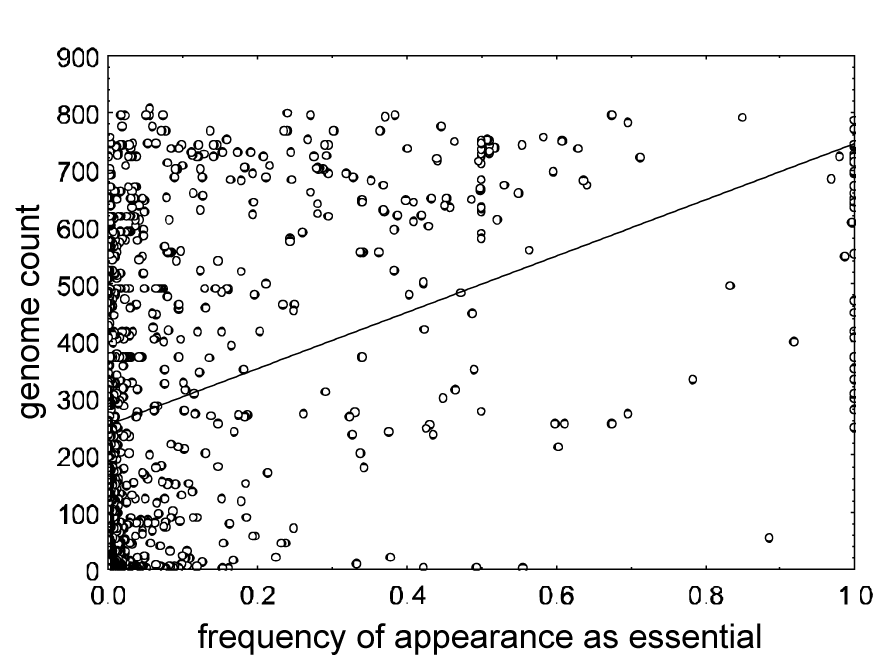

Supplement: Figure S4 — Reaction essentiality and gene appearance in prokaryotic genomes. Correlation of frequency of reaction essentiality in random metabolic networks and number of genomes carrying an enzyme-coding gene catalyzing that reaction. Pearson's r = 0.45; p = 2.2×10^−16^. This analysis uses enzyme-coding genes from 875 prokaryotic genomes in the KEGG database (0.10 MB TIF) [file pcbi.1000613.s005.tif]

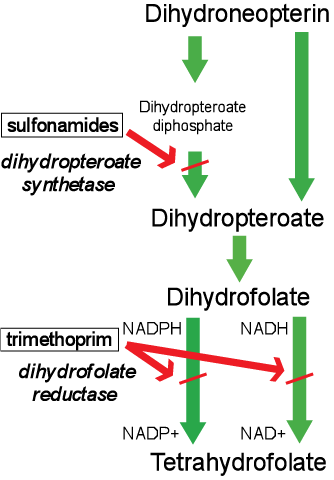

Supplement: Figure S5 — Reactions in tetrahydrofolate biosynthesis and their essentiality. We found that the reaction dihydropteroate synthetase, a target of sulfonamides, is essential in 41% of the metabolic networks we studied, while the other reaction producing dihydropteroate is essential in 56.1% of networks. In the remaining 2.9% of networks, both reactions appear, but none are essential. These observations have a straightforward explanation. Dihydropteroate is an essential metabolite. Because only two alternative reactions exist to make dihydropteroate, whenever one of these reactions is missing, the other is an essential reaction. Whenever both reactions are present, neither reaction is essential. For the production of tetrahydrofolate from dihydrofolate, there exist, similarly, two parallel dihydrofolate reductase reactions. These reactions are the target of trimethoprim. The reactions are only distinguished by the molecule that acts as the electron donor, either NADH or NADPH. Individually, these reactions appear as essential in only 30%–40% of networks. In addition, only 66.2% of networks cannot tolerate the removal of both reactions. The reason is that there are alternative paths (not shown) that bypass the direct production of tetrahydrofolate from dihydrofolate. (0.06 MB TIF) [file pcbi.1000613.s006.tif]

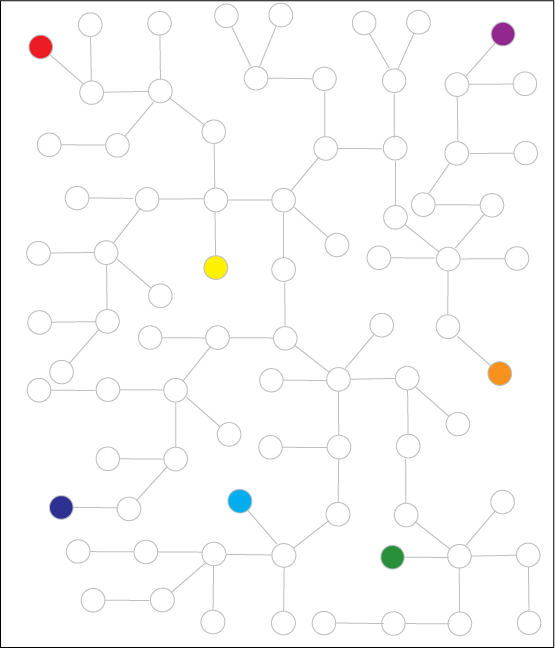

Supplement: Figure S6 — The connectedness of metabolic networks with the same phenotype facilitates access to new metabolic phenotypes. The rectangle symbolizes genotype space, and the grey circles symbolize metabolic networks with a given metabolic phenotype. The colored circles stand for metabolic networks with a novel phenotype. Different novel phenotypes (different colors) are accessible from different networks (points) in genotype space with the same phenotype. (0.08 MB TIF) [file pcbi.1000613.s007.tif]
